# Supplementary material for: Unexpected mutual regulation underlies paralogue functional diversification and promotes epithelial tissue maturation in Tribolium
Source: Commun Biol. 2020 Oct 5;3:552. doi: 10.1038/s42003-020-01250-3 (PMC7536231; doi:10.1038/s42003-020-01250-3)
Supplement: Supplementary file 1 — Supplementary Information [file 42003_2020_1250_MOESM1_ESM.pdf]

Unexpected mutual regulation underlies paralogue functional diversification and promotes epithelial tissue maturation in *Tribolium*

Supplementary Information

<u>Within this PDF</u>:

Supplementary Figure 1. Alignments of *zen1* and *zen2* homeoboxes/domains.

Supplementary Figure 2. Additional phylogenies of insect class 3 Hox proteins.

Supplementary Figure 3. Phenotypic consequences of *Tc-zen2* RNAi - milder defects.

Supplementary Figure 4. Tc-Zen1 and Tc-Zen2 expression detected by western blotting.

Supplementary Figure 5. Early *in vivo* expression dynamics of transcript and protein for the *Tc-zen* paralogues.

Supplementary Figure 6. Expression screen summary of early Tc-Zen2 candidate targets.

Supplementary Figure 7. Gene ontology (GO) enrichment analysis after *Tc-zen2* RNAi.

Supplementary Figure 8. Functional annotation and validation of late Tc-Zen2 targets.

Supplementary Table 1. List of gene ontology (GO) terms grouped by category of interest in each GO domain.

Supplementary Table 2. Description of Tc-Zen2 late candidate target genes validated by RT-qPCR.

Supplementary Table 3. *Tribolium castaneum* (*TC*) primer sequences for *in situ* hybridization, RNAi, and RT-qPCR.

Supplementary Table 4. Comparison of alignment statistics by read length.

Other supplementary files:

(Excel workbook files with large datasets, gene lists, source values)

Supplementary Data 1. Tables of differentially expressed genes during early development (P_{adj}≤0.01). **(A)** After *Tc-zen1* RNAi compared to wild type, at 6-10 hAEL. **(B)** After *Tc-zen2* RNAi compared to wild type, at 10-14 hAEL. **(C)** Between wild type at 6-10 hAEL and 10-14 hAEL.

Supplementary Data 2. Regulatory targets shared by both *Tc-zen* paralogues with relaxed thresholds (P_{adj}≤0.05). **(A)** Same direction of regulation, n=42. **(B)** Opposite direction of regulation, n=78.

Supplementary Data 3. Tables of differentially expressed genes during late development (P_{adj}≤0.01). **(A)** After *Tc-zen2* RNAi compared to wild type, at pre-rupture (48-52 hAEL). **(B)** After *Tc-zen2* RNAi compared to wild type, during rupture (52-56 hAEL). **(C).** Between wild type at 48-52 hAEL and 52-56 hAEL. **(D)** Between *Tc-zen2* RNAi at 48-52 hAEL and 52-56 hAEL. **(E)** Between wild type at 48-52 hAEL and *Tc-zen2* RNAi 52-56 hAEL.

Supplementary Data 4. Tables of enriched gene ontology (GO) terms for differentially expressed genes after *Tc-zen2* RNAi in late development. **(A)** At pre-rupture (48-52 hAEL). **(B)** During rupture (52-56 hAEL).

Supplementary Data 5. Source values and dual plotting (means, individual values) for bar charts in figures.

Supplementary Figures

```
A        1                                   10                                      20
                                                                  HELIX 1
         G   K   R   A   R   T   A   Y   T   S   A   Q   L   V   E   L   E   R   E   F
         G   K   R   A   R   T   A   Y   T   S   A   Q   L   V   E   L   E   R   E   F
         G   K   R   A   R   T   A   Y   T   S   A   Q   L   V   E   L   E   R   E   F
         P   K   R   V   R   T   A   Y   T   S   A   Q   L   V   E   L   E   R   E   F
Tcas-z1  GGA AAG CGT GCC CGC ACT GCC TAC ACC TCG GCC CAA TTA GTG GAA TTA GAG CGG GAA TTC
Tfre-z1  GGA AAA CGT GCT CGC ACT GCT TAC ACC TCA GCC CAG TTA GTG GAA TTA GAG CGG GAA TTC
Tmad-z1  GGA AAA CGT GCT CGC ACT GCG TAT ACC TCG GCT CAA TTA GTC GAA TTA GAG CGG GAA TTT
Tcon-z1  CCA AAA CGT GTC CGC ACT GCT TAC ACA TCA GCC CAA TTA GTA GAA CTA GAA CGG GAA TTC
           * **  *** *   *** *** **  **  **  **  **  **  *** **  ***  ** **  *** *** **

         21                                  30                                      40
                                                   HELIX 2
         H   H   G   K   Y   L   S   R   P   R   R   I   Q   I   A   E   N   L   N   L
         H   L   G   K   Y   L   S   R   P   R   R   I   Q   M   A   E   N   L   N   L
         H   R   G   K   Y   L   T   R   P   R   R   I   Q   M   A   E   N   L   K   L
         H   R   G   K   Y   L   S   R   P   R   R   I   Q   M   A   T   N   L   N   L
Tcas-z1  CAC CAC GGG AAA TAC TTA TCA CGA CCA CGC CGA ATC CAA ATC GCC GAG AAC CTG AAC CTG
Tfre-z1  CAT CTC GGG AAA TAT TTA TCA CGG CCG CGA CGA ATC CAA ATG GCC GAA AAT CTA AAC CTG
Tmad-z1  CAT CGC GGG AAA TAT TTA ACA CGG CCG CGA AGA ATC CAA ATG GCT GAA AAT TTG AAA CTG
Tcon-z1  CAT CGT GGA AAA TAT TTA TCA CGA CCT AGG AGA ATC CAG ATG GCC ACA AAT CTA AAT CTC
         **  *   **  *** **  ***  ** **  **   *   ** *** **  **  **      **   *  **  **

         41                                  50                                      60
                                      HELIX 3
         S   E   R   Q   I   K   I   W   F   Q   N   R   R   M   K   H   K   K   E   Q
         S   E   R   Q   I   K   I   W   F   Q   N   R   R   M   K   Y   K   K   E   Q
         S   E   R   Q   I   K   I   W   F   Q   N   R   R   M   K   H   K   K   E   Q
         S   E   R   Q   I   K   I   W   F   Q   N   R   R   M   K   H   K   K   E   Q
Tcas-z1  AGT GAG CGT CAG ATC AAG ATT TGG TTC CAA AAT CGC AGA ATG AAG CAC AAG AAA GAA CAG
Tfre-z1  AGT GAG CGT CAA ATC AAA ATT TGG TTC CAG AAC CGA AGA ATG AAG TAC AAG AAG GAG CAG
Tmad-z1  AGT GAA CGT CAA ATC AAA ATT TGG TTC CAA AAT CGA AGA ATG AAG CAC AAG AAA GAA CAG
Tcon-z1  AGT GAA CGT CAA ATC AAG ATT TGG TTT CAA AAT CGC AGA ATG AAG CAC AAG AAG GAG CAA
         *** **  *** **  *** **  *** *** **  **  **  **  *** *** ***  ** *** **  **  **
```

```
B        1                                   10                                      20
                                                                  HELIX 1
         G   K   R   A   R   T   A   Y   T   S   S   Q   L   V   E   L   E   R   E   F
Tcas-z2  GGC AAA AGG GCC CGA ACT GCG TAC ACC TCC TCC CAA TTA GTC GAA CTC GAA CGC GAA TTT
Tfre-z2  GGC AAA AGG GCC CGA ACT GCA TAC ACC TCC TCT CAA TTA GTC GAA CTG GAA CGC GAA TTT
Tmad-z2  GGC AAA AGA GCC CGA ACT GCC TAC ACC TCC TCA CAA TTA GTA GAA TTA GAA CGC GAA TTT
Tcon-z2  GGA AAA AGA GCT CGA ACT GCA TAC ACT TCC TCT CAA TTA GTC GAA TTA GAG CGC GAA TTC
         **  *** **  **  *** *** **  *** **  *** **  *** *** **  ***  *  **  *** *** **

         21                                  30                                      40
                                                   HELIX 2
         H   R   S   K   Y   L   C   R   P   R   R   I   Q   M   A   Q   N   L  N/S  L
Tcas-z2  CAC CGG AGC AAG TAC TTG TGC CGC CCT CGG CGC ATC CAA ATG GCC CAA AAC CTC AAC TTG
Tfre-z2  CAC CGC AGT AAA TAC TTA TGT CGA CCT CGG CGC ATM CAA ATG GCC CAA AAT CTC AAC TTG
Tmad-z2  CAT CGA AGC AAA TAT TTA TGT CGT CCT CGT CGT ATT CAA ATG GCC CAA AAC CTC AAT TTA
Tcon-z2  CAC CGT AGC AAG TAT TTA TGT AGA CCT CGT CGC ATC CAA ATG GCT CAA AAT CTA AGC CTT
         **  **  **  **  **  **  **   *  *** **  **  **  *** *** **  *** **  **  *    *

         41                                  50                                      60
                                      HELIX 3
         T   E   R   Q   I   K   I   W   F   Q   N   R   R   M   K   F   K   K   E   E
Tcas-z2  ACC GAA CGC CAA ATC AAG ATT TGG TTC CAA AAC CGC AGA ATG AAG TTC AAA AAG GAG GAG
Tfre-z2  ACT GAG CGT CAA ATC AAG ATT TGG TTC CAA AAC CGT AGA ATG AAG TTC AAA AAG GAG GAA
Tmad-z2  ACT GAA CGC CAA ATA AAA ATT TGG TTT CAA AAC CGA CGT ATG AAG TTC AAG AAA GAA GAA
Tcon-z2  ACT GAA CGC CAA ATC AAA ATT TGG TTT CAA AAT CGT CGC ATG AAA TTC AAA AAA GAA GAA
         **  **  **  *** **  **  *** *** **  *** **  **   *  *** **  *** **  **  **  **
```

codons with non-synonymous substitutions   M A or C (nucleotide sequence ambiguity)

Nucleotide substitution at: first position second position third position

Supplementary Fig. 1. Alignments of *zen1* and *zen2* homeoboxes/domains. The alignments of *zen1* **(A)** and *zen2* **(B)** homeoboxes/domains of four *Tribolium* species (*T. castaneum* - *Tcas*; *T. freeman* - *Tfre*; *T. madens* - *Tmad*; *T. confusum* - *Tcon*) show that the number of non-synonymous substitutions is higher in the Zen1 homeodomain (9 substitutions) than in the Zen2 homeodomain (1; see also Fig. 1B, 1C).

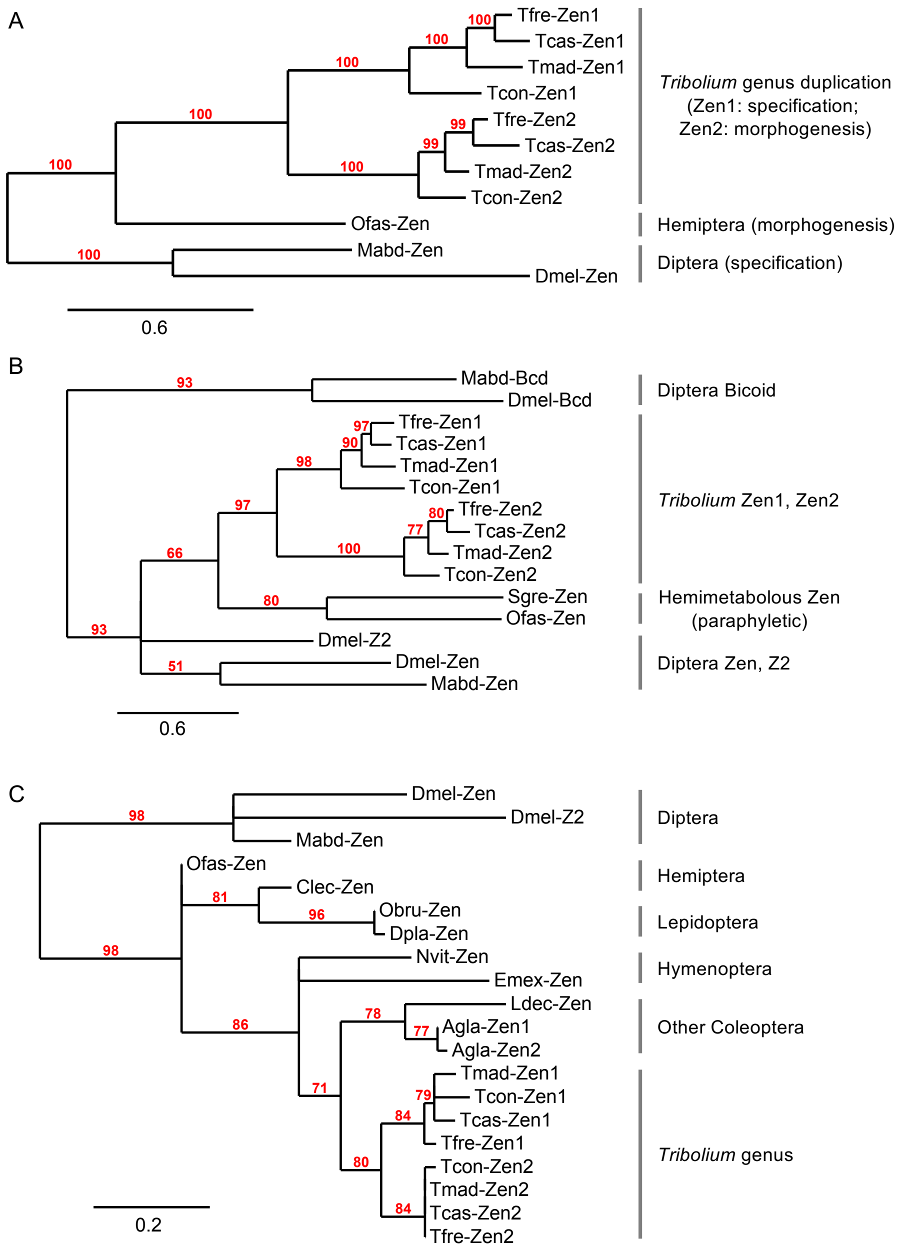

Supplementary Fig. 2. Additional phylogenies of insect class 3 Hox proteins (Zen and Bicoid orthologues and paralogues). Phylogenetic trees that differ in taxa, gene class, protein sequence input, and phylogenetic method consistently support an origin of the *zen* tandem duplication within the *Tribolium* genus of the Coleoptera. Taxon and protein sampling choice is for Zen proteins with known extraembryonic functions **(A)**, insect class 3 Hox genes with known expression patterns **(B)**, and wider sampling for taxonomic diversity based on sequence data alone (**C**, two species from each of five insect orders). All *Tribolium* species are included in all analyses. Trees were generated by: Bayesian methods on full-length Zen and Bicoid proteins (A-B), or maximum likelihood with the homeodomain alone (C; compare with full-length proteins and maximum likelihood in Fig. 1B); see main text methods for further details. In total, 38 trees with different parameters and taxa (30 proteins from 21 arthropod species) were analyzed, with consistent results.

Results: Across the trees shown here and Fig. 1B, *Tribolium* Zen proteins form a well-supported clade (≥76% node support), with subclades for each of Zen1 and Zen2 (≥84%). The topology within each paralogue's subclade also parallels the species tree (Fig. 1A), except where there is a loss of resolution in (C), which does not examine full-length proteins. However, rapid sequence divergence and long branch attraction consistently result in dipteran sequences falling as molecular outgroups, and with

deviations between the gene trees and species tree relationships at the ordinal level and for more ancient divergence times.

Abbreviations and sequence sources (GenBank accessions unless otherwise noted): Agla, *Anoplophora glabripennis* independent Zen duplicates (OGS v1.2: Zen1, AGLA004144-RA; Zen2, AGLA014266-RA)[1]; Clec, *Cimex lectularius* (OGS v1.2: CLEC001685-RA)[2]; Dmel, *Drosophila melanogaster* Zen (NP_476793.1), Z2 (NP_476794.1), Bicoid (NP_731111.1); Dpla, *Danaus plexippus plexippus* (OWR47614.1); Emex, *Eufriesea mexicana* (XP_017753571); Ldec, *Leptinotarsa decemlineata* (OGS v1.2: LDEC004404-PA)[3]; Mabd, *Megaselia abdita* Zen (CAB40893.1), Bicoid (CAB40892.1); Nvit, *Nasonia vitripennis* (XP_001603758.2); Obru, *Operophtera brumata* (KOB75729.1); Ofas, *Oncopeltus fasciatus* (ABC17998.1, and OGS v1.2: OFAS000840-RA); Sgre, *Schistocerca gregaria* (CAB61208.1); *Tribolium* species as in the main text. Where protein IDs are from a specified OGS version, these gene models are available at https://i5k.nal.usda.gov/, last accessed 27 February 2020.

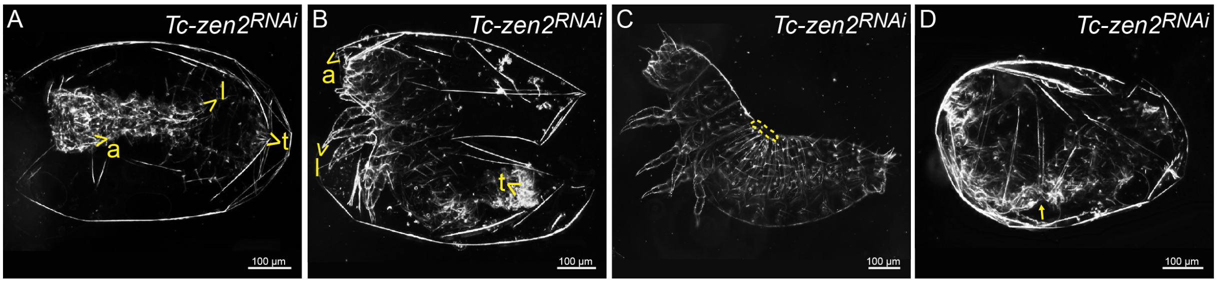

Supplementary Fig. 3. Phenotypic consequences of *Tc-zen2* RNAi – milder defects, based on larval cuticle preparations. (A) Partially everted phenotype - anterior inside out. **(B)** Partially everted phenotype - posterior inside out. **(C)** Dorsal open (small medial hole within dashed circle). **(D)** Cuticle crumbs - larval cuticle is constricted in the middle (arrow), presumably due transient contractile squeezing by the EEMs after a loss in tissue integrity[4]. Anatomical abbreviations: a, antenna; l, limb; t, telson. Scale bars are 100 µm. Frequencies of these phenotypic classes are summarized in Fig. 2F.

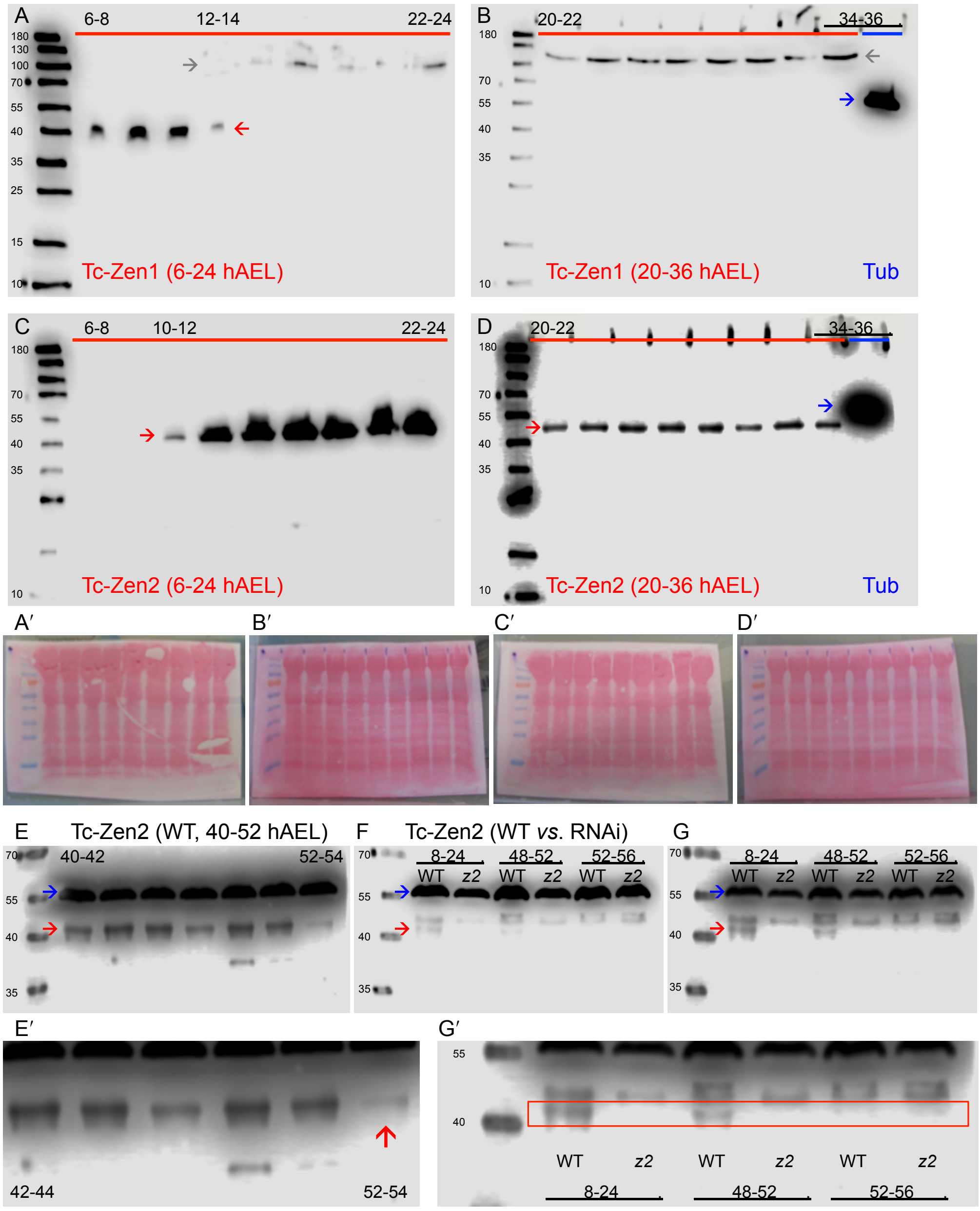

Supplementary Fig. 4. Tc-Zen1 and Tc-Zen2 expression detected by western blotting. Images presented here show additional blots in support of Fig. 4A. **(A-D)** Early through mid development expression of Tc-Zen1 (A-B) and Tc-Zen2 (C-D), sampled in two-hour intervals from blastoderm through extended germband stages (A, C: 6-24 hAEL), and extended germband through mid germband retraction stages (B, D: 20-36 hAEL, note overlap of staging between left and right blots). In these samples, the membrane was cut vertically and the strongly expressed anti-Tubulin control (59.0 kDa, blue arrows, "Tub") is only detected in the rightmost lane of the mid-embryogenesis blots (B, D). Both Tc-Zen1 and Tc-Zen2 are cleanly detected as single bands (red arrows). The background signal >100 kDa (grey arrows) in the Tc-Zen1 blots (A-B) is attributed to debris associated with the development of the serosal cuticle (from 14 hAEL) in these whole-egg preparations. **(A′-D′)** Ponceau S staining to visualize whole-egg lysate material. **(E-E′)** Late development expression of Tc-Zen2 (40-54 hAEL). Although Tc-Zen2 expression drops from 50-52 hAEL (pre-rupture), its expression is still detected during membrane withdrawal (arrow at 52-54 hAEL in the enlarged inset image in E′), but it can only be observed on high intensity images (same blot as in Fig. 4A, here shown at higher intensity). **(F-G′)** To confirm RNAi knockdown for *Tc-zen2*, we compared wild type (WT) and RNAi

samples (*z2*) at selected stages: across a broad interval of early development when wild type Tc-Zen2 is strongly expressed (8-24 hAEL), and at each of the stages chosen for the late RNA-seq analysis (pre-rupture, 48-52 hAEL; during withdrawal, 52-56 hAEL). The same blot is shown at low (F) and high (G) intensity. Red and blue arrows indicate Tc-Zen2 and anti-Tubulin, respectively. Note the absence of expression of Tc-Zen2 in the RNAi samples, highlighted within the red-boxed region in the high magnification inset in G′. However, as wild type Tc-Zen2 is very weakly expressed from 52 hAEL (E), both wild type and RNAi samples show an absence of Tc-Zen2 at the 52-56 hAEL stage. Nonetheless, as the 52-56 hAEL samples were obtained within the same experiments as the 48-52 hAEL samples, we considered these samples to represent valid RNAi knockdown material, a fact borne out in the subsequent RNA-seq analyses. The bands immediately above the Tc-Zen2 band do not vary by stage or treatment (relative to the anti-Tubulin control) and are therefore interpreted as unspecific background. Left-hand size standards are in kDa (all bands labeled in A, selected bands labeled in subsequent blots).

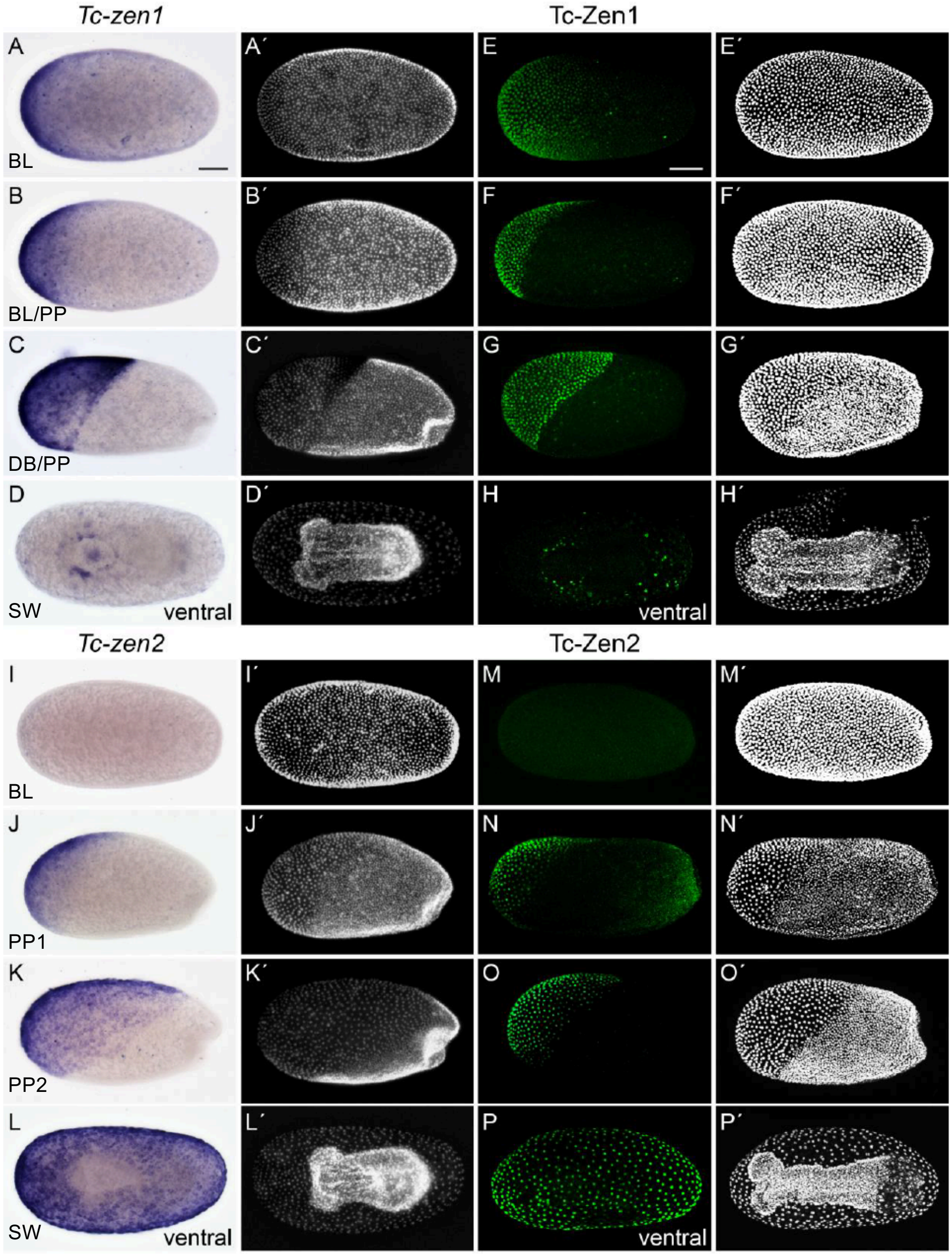

Supplementary Fig. 5. Early *in vivo* expression dynamics of transcript and protein for the *Tc-zen* paralogues. **(A-D)** *Tc-zen1 in situ* hybridization. **(E-H)** Tc-Zen1 immunohistochemistry. **(I-L)** *Tc-zen2 in situ* hybridization. **(M-P)** Tc-Zen2 immunohistochemistry. Whole mount eggs are shown from the blastoderm through serosal window closure stages, in support of Figs. 3-4, using the same *in situ* probes and paralogue-specific peptide antibodies[5] (panels A, I, J, P are reproductions from Figs. 3B, 3G, 3H, and 4C, respectively). Letter-prime images show a nuclear counterstain (DAPI). Images are oriented with anterior left, in lateral aspect with dorsal up unless otherwise indicated or for uniform blastoderm stages. Scale bars are 100 µm (A, E) and apply to all respective *in situ* and immunohistochemistry images and their counterstains. Staging abbreviations: BL, blastoderm formation/ uniform blastoderm; DB, differentiated blastoderm; PP, primitive pit; SW, serosal window. Overall, there is a temporal offset whereby *Tc-zen1* precedes *Tc-zen2*. For both genes, protein expression (and nuclear localization) immediately follows transcript expression. By the serosal window stage, *Tc-zen1* has largely switched off (D, H), while *Tc-zen2* has expanded throughout the serosa (L, P).

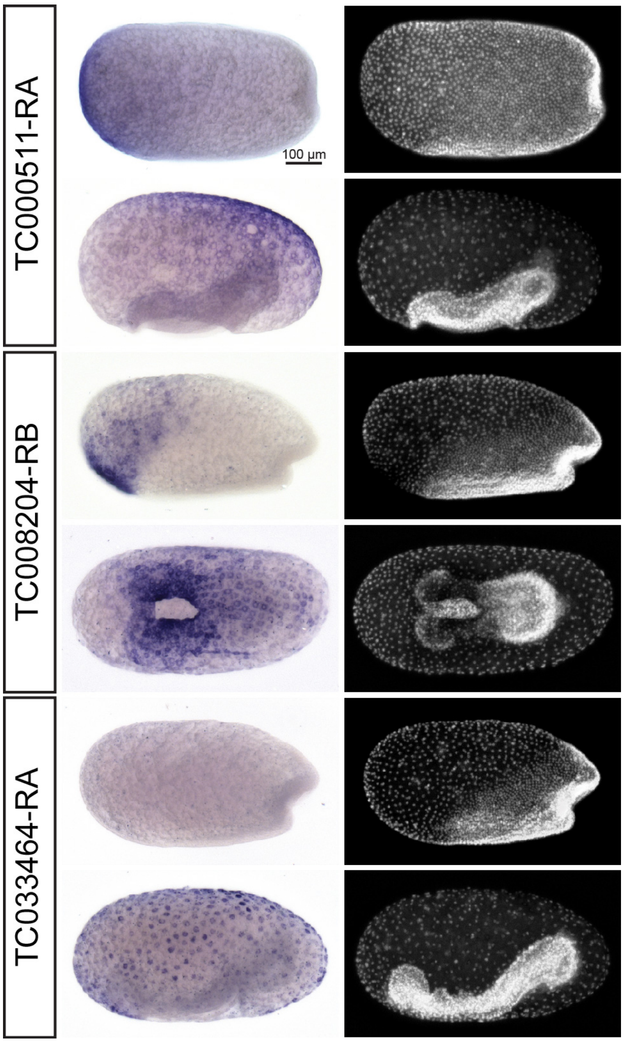

Supplementary Fig. 6. Expression screen summary of early Tc-Zen2 candidate targets. Genes were chosen based on RNA-seq after RNAi differential expression ($P_{adj} \leq 0.01$, $|\log2\ FC| \geq 1$, at 10-14 hAEL; Supplementary Data 1B), with 3 of 19 tested genes shown here. For each gene, expression was evaluated by *in situ* hybridization during early and late EEM formation: "primitive pit" (upper image) and "serosal window" (lower image) stages, respectively, with staging determined by DAPI counterstain (right images). Early developmental dynamics mean that these three validated candidates, all with specific serosal expression, include factors that are either upregulated or downregulated by Tc-Zen2, and with distinct staging for the onset of expression. All views are lateral except for a ventral view in the lower panel for *TC008204-RB*; scale bar is 100 µm (applies to all images).

TC000511-RA (log2 FC -1.8074; *Drosophila* homologue *hadley*, peak expression during pupal stages: see comment on imaginal disc morphogenesis in Supplementary Fig. 8) expression starts in the anterior of the serosa. During serosal expansion the expression spreads into the whole serosa, although transcript levels then appear to decline in the most anterior part.

TC008204-RB (log2 FC 1.2187; *Arylalkylamine N-acetyltransferase 1, aaNAT*, cuticle sclerotization factor) expression begins with highest transcript levels detected in the anterior-ventral region of the serosa. This gene is activated by Tc-Zen1 before being repressed by Tc-Zen2 (see Figs. 5B, 6I), and this is reflected in the contracting expression domain centered around the serosal rim at the later stage, similar to but broader than the domain of *Tc-zen1* at this stage (*cf.*, Figs. 3D-F, 6E).

TC033464-RA (log2 FC -2.7879; *Drosophila* CG13618 homologue, kinase domain) transcript is not detected until the serosal window stage, when perinuclear expression is observed throughout the tissue, consistent with this being a later Tc-Zen2 target during serosal maturation.

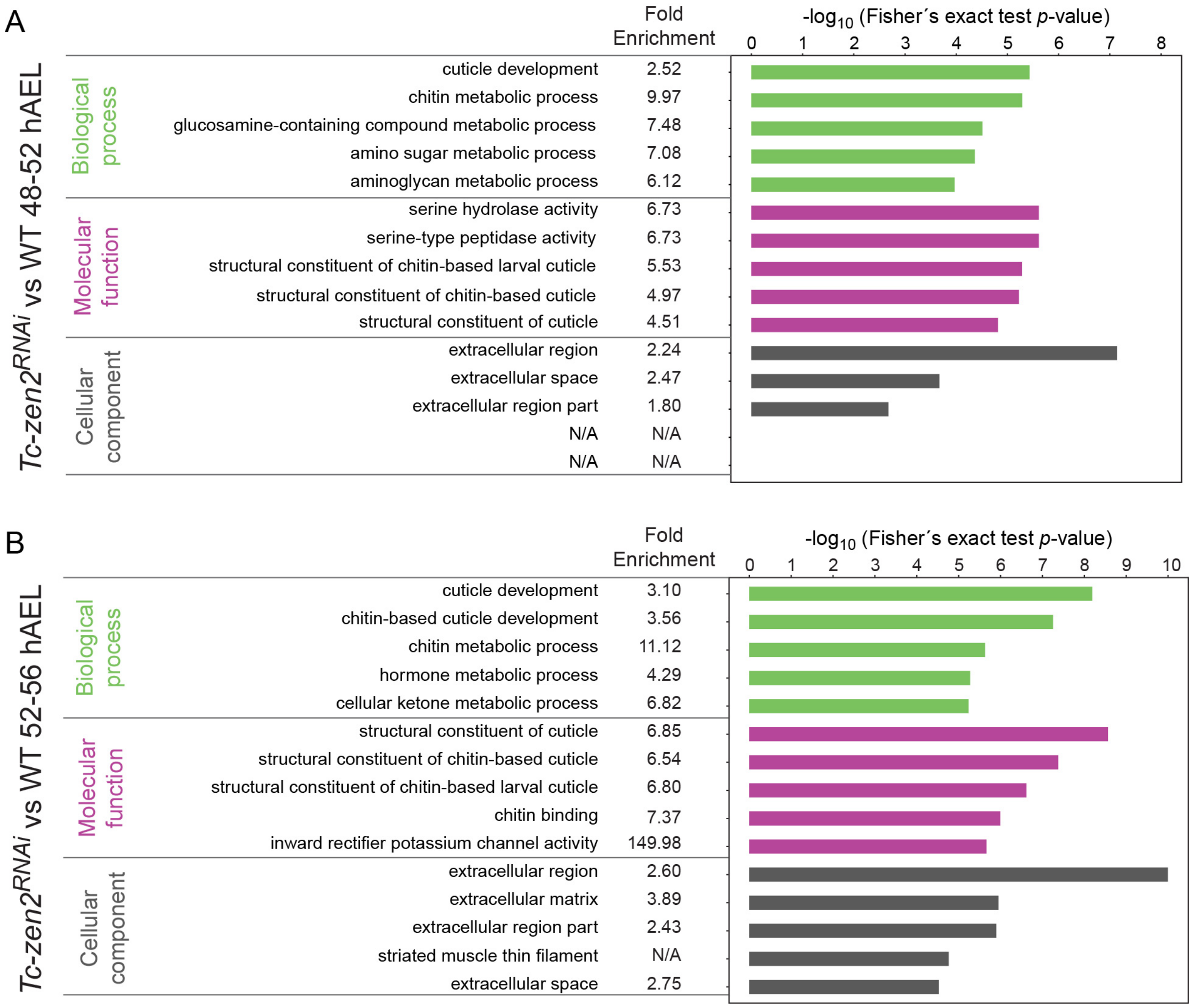

Supplementary Fig. 7. Gene ontology (GO) enrichment analysis of the differentially expressed genes in the pre-rupture **(A)** and rupture/post-rupture **(B)** stages after *Tc-zen2* RNAi. If applicable, the five most significantly enriched GO terms and their fold enrichment per GO domain are shown for each of the datasets (Fisher´s exact test, two-sided, threshold false discovery rate (FDR) of 0.05). Only over-represented GO terms are shown.

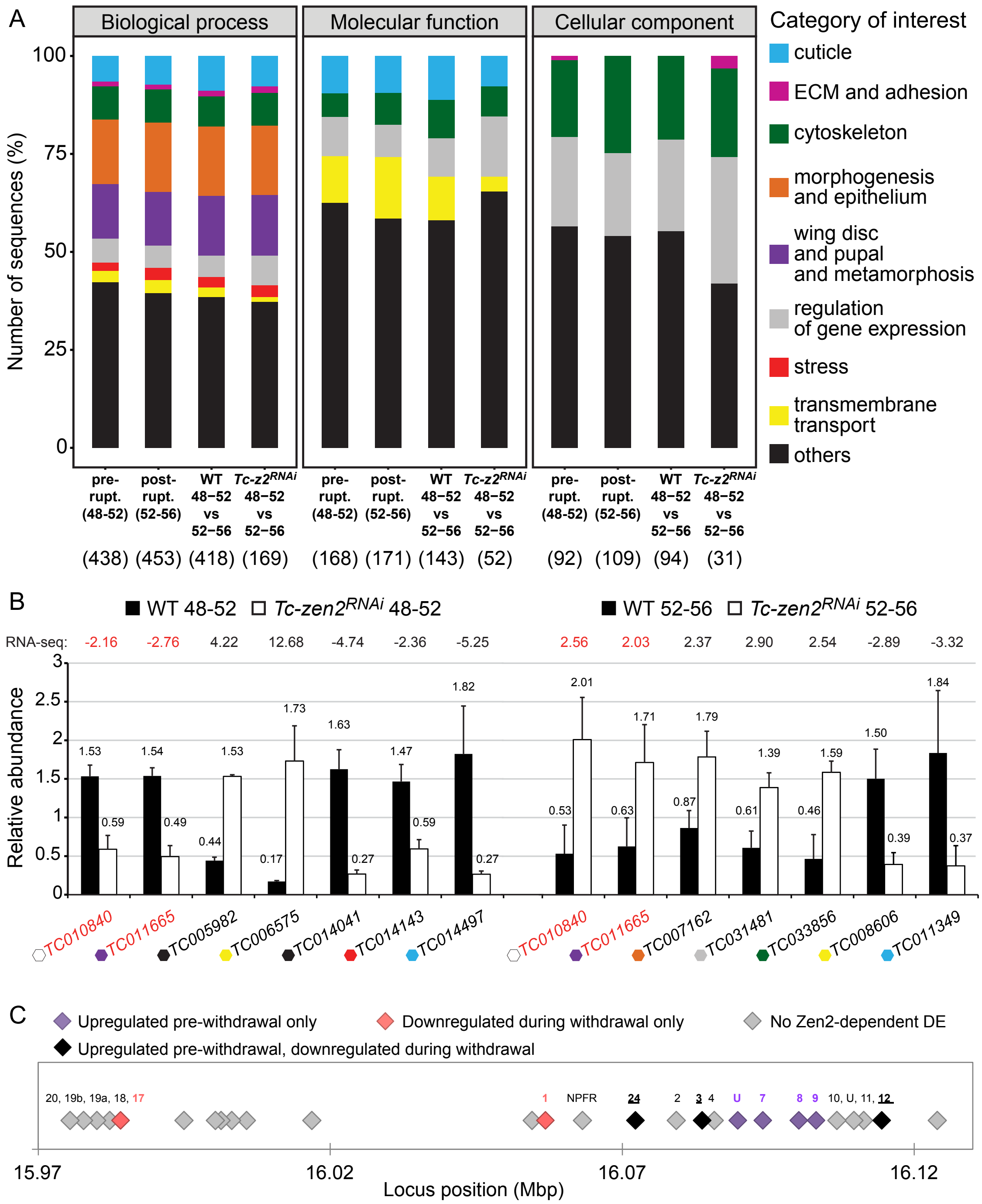

Supplementary Fig. 8. Functional annotation and validation of late developmental targets of Tc-Zen2. **(A)** GO profiles per pairwise DE comparison (comparisons as in Fig. 5D). Our GO categories reflect known serosal features (note legend; see main text Methods, Supplementary Table 1). In addition to cytoskeletal, epithelial, and extracellular remodeling, we considered *Drosophila* imaginal disc morphogenesis, which has noted similarities to EEM withdrawal[4]. We included transcriptional regulation, as *Tc-zen2* may act upstream in serosal gene regulatory networks. We also looked for stress response genes, given the unnatural constraint of the embryo in the absence of EEM withdrawal[6]. These categories account for ~60%, ~35%, and ~50% of sequences for the respective categories biological process, molecular function, and cellular component. Transmembrane transport proteins comprise an additional, prominent category that we had not deliberately selected (yellow). Transporters may serve physiological roles in the serosa as a barrier epithelium, with the potential to mediate exchange between the egg and the outer environment and for yolk catabolism[7-9]. Numbers are

GO domains per DE comparison. Note that a given gene may be assigned to multiple GO categories, depending on available information in public databases. **(B)** RT-qPCR testing of selected Tc-Zen2 candidate targets. Candidates were chosen from the GO categories in A (note color-coding, where white indicates a novel gene; see also Supplementary Table 2) and based on the direction of Tc-Zen2 regulation. Seven candidates were chosen from each stage (48-52 hAEL and 52-56 hAEL), where two of these show changing direction of regulation across stages (red text). All RNA-seq DE predictions (fold change values above the graph) were validated by RT-qPCR (mean value from three biological replicates; error bars represent one standard deviation; for clarity, mean values are stated here, with the individual source data values given in Supplementary Data 5). **(C)** Mapping of Tc-Zen2-dependent regulation to genes within the *Osiris* complex of tandemly duplicated, co-expressed *Osiris* genes (shown for a portion of scaffold NC_007424.3, based on Official Gene Set v3 (OGS3), gene locus positions as defined in http://ibeetle-base.uni-goettingen.de, last accessed 5 August 2019). All predicted genes are represented by diamonds, with the *Osiris* gene homology indicated above *Osiris* family genes (U: uncertain homology) and for the conserved, syntenic factor NPFR[10], while unrelated genes are unlabeled. Notably, although the *Osiris* genes are co-expressed and thus represent an exemplar locus of open, accessible chromatin for co-regulation, we detect Tc-Zen2-dependent regulation only for a subset of this gene family. Moreover, whereas some tandem genes are co-regulated by Tc-Zen2 (purple: *Osiris genes U/7/8/9*), other genes that are co-regulated and show changing Tc-Zen2-dependent regulation occur throughout the gene complex (black: *Osiris 24, 3, 12*). As detailed in Table S6, gene TC011665, validated by RT-qPCR in panel B, is *Osiris 12*. Regulation color-coding is as in Fig. 5E. Staging is the same as in the rest of the figure (48-52 hAEL: pre-withdrawal; 52-56 hAEL: during withdrawal morphogenesis).

Supplementary Tables

Supplementary Table 1. List of gene ontology (GO) terms grouped by category of interest in each GO domain.

Category of interest	GO domain: Biological process
Stress	response to endoplasmic reticulum stress
	cellular response to oxidative stress
	regulation of translation in response to stress
	regulation of response to osmotic stress
	stress-activated protein kinase signaling cascade
	positive regulation of stress fiber assembly
	age-dependent response to oxidative stress
	regulation of stress fiber assembly
Cuticle	chitin-based cuticle development
	molting cycle, chitin-based cuticle
	cuticle pigmentation
	chitin-based cuticle sclerotization
	ecdysis, chitin-based cuticle
	regulation of adult chitin-containing cuticle pigmentation
	regulation of chitin-based cuticle tanning
	cuticle pattern formation
Cytoskeleton	cytoskeleton organization
	actin cytoskeleton organization
	actin filament organization
	actin filament bundle assembly
	cytoskeleton-dependent cytokinesis
	actin filament-based movement
	regulation of actin cytoskeleton organization
	establishment or maintenance of cytoskeleton polarity
	cytoskeleton-dependent intracellular transport
	microtubule cytoskeleton organization involved in mitosis
	cytoskeletal anchoring at plasma membrane
	oocyte microtubule cytoskeleton organization
	establishment or maintenance of microtubule cytoskeleton polarity
	regulation of actin filament length
Epithelium and morphogenesis	epithelium development
	morphogenesis of an epithelium
	epithelial tube morphogenesis
	cell morphogenesis
	cell part morphogenesis
	cell projection morphogenesis
	imaginal disc morphogenesis
	post-embryonic animal organ morphogenesis
	imaginal disc-derived appendage morphogenesis
	post-embryonic appendage morphogenesis
	cell morphogenesis involved in differentiation
	sensory organ morphogenesis
	epithelial cell development
	gland morphogenesis
	digestive tract morphogenesis
	epithelium migration
	regulation of organ morphogenesis
	Malpighian tubule morphogenesis
	dendrite morphogenesis
	morphogenesis of embryonic epithelium
	embryonic hindgut morphogenesis
	regulation of cell morphogenesis
	regulation of morphogenesis of an epithelium
	antennal morphogenesis
	trachea morphogenesis
	epithelial cell proliferation involved in renal tubule morphogenesis
	epithelial tube formation
	mesoderm morphogenesis
	transepithelial transport
	spermathecum morphogenesis

	heart morphogenesis
Epithelium and morphogenesis	branching morphogenesis of an epithelial tube
	negative regulation of cell morphogenesis involved in differentiation
	morphogenesis of a branching epithelium
	chaeta morphogenesis
	regulation of cell proliferation involved in imaginal disc-derived wing morphogenesis
	male genitalia morphogenesis
	imaginal disc-derived leg joint morphogenesis
	intestinal epithelial cell differentiation
	positive regulation of cell morphogenesis involved in differentiation
	post-embryonic genitalia morphogenesis
	cell elongation involved in imaginal disc-derived wing morphogenesis
Wing disc and pupal and metamorphosis	instar larval or pupal development
	wing disc development
	instar larval or pupal morphogenesis
	metamorphosis
ECM and adhesion	cell-matrix adhesion
	extracellular matrix organization
	negative regulation of cell-cell adhesion
Regulation of gene expression	gene expression
	regulation of gene expression
	negative regulation of gene expression
	positive regulation of gene expression
	regulation of translational initiation
	chromatin silencing
	negative regulation of chromatin silencing
	regulation of chromatin silencing
Transmembrane transport	transmembrane transport
	regulation of transmembrane transport
	regulation of transmembrane transporter activity
	positive regulation of ion transmembrane transporter activity
	positive regulation of ion transmembrane transport
**Category of interest**	**GO domain: Molecular function**
Regulation of gene expression	DNA binding
	transcription factor activity, RNA polymerase II distal enhancer sequence-specific binding
	transcription factor activity, RNA polymerase II core promoter proximal region sequence-specific binding
	transcriptional activator activity, RNA polymerase II transcription regulatory region sequence-specific binding
	transcriptional repressor activity, RNA polymerase II transcription regulatory region sequence-specific binding
	flavin adenine dinucleotide binding
	purine ribonucleotide binding
	purine nucleoside binding
	purine nucleotide binding
	ribonucleoside binding
	purine ribonucleoside triphosphate binding
	RNA binding
	repressing transcription factor binding
	regulatory region nucleic acid binding
	chromatin insulator sequence binding
Cuticle	structural constituent of chitin-based larval cuticle
	chitin deacetylase activity
Cytoskeleton	myosin binding
	actin binding
	actinin binding
	actin filament binding
Transmembrane transport	ion transmembrane transporter activity
	secondary active transmembrane transporter activity
	primary active transmembrane transporter activity
	organic acid transmembrane transporter activity
	sulfate transmembrane transporter activity

	active ion transmembrane transporter activity
Transmembrane transport	monoamine transmembrane transporter activity
	taurine transmembrane transporter activity
	phosphate ion transmembrane transporter activity
	serotonin transmembrane transporter activity
**Category of interest**	**GO domain: Cellular component**
Regulation of gene expression	nucleus
	chromatin
	nuclear chromosome part
	nuclear chromosome
	chromosomal region
	nuclear transcription factor complex
Cytoskeleton	cytoskeleton
	contractile fiber
	unconventional myosin complex
	polymeric cytoskeletal fiber
	cell cortex region
	cell cortex part
	myosin II complex
ECM and adhesion	focal adhesion

Supplementary Table 2. Description of Tc-Zen2 late candidate target genes validated by RT-qPCR. *Dmel* - *Drosophila melanogaster*; FC - fold change. See Supplementary Fig. 8B.

gene ID	developmental stage	GO descriptor	*Dmel* homologue	FC
TC010840	pre-rupture<br>post-rupture	no information available;<br>DUF4773 (pfam 15998)	no homologue	-2.16<br>2.56
TC011665	pre-rupture<br>post-rupture	morphogenesis and epithelia (imaginal disc-derived wing morphogenesis);<br>DUF1676 (pfam 07898)	Osiris 12	-2.76<br>2.03
TC005982	pre-rupture	cytosol (other)	Major facilitator superfamily transporter 3	4.22
TC006575	pre-rupture	transmembrane transport	Pickpocket 26	12.68
TC014041	pre-rupture	cell differentiation (other)	Z band alternatively spliced PDZ-motif	-4.74
TC014143	pre-rupture	stress	Protein kinase, cAMP-dependent, catalytic subunit 3	-2.36
TC014497	pre-rupture	cuticle	Cuticular protein 65Av	-5.25
TC007162	post-rupture	morphogenesis and epithelia	Ribbon	2.37
TC031481	post-rupture	regulation of gene expression	Nuclear factor interleukin-3-regulated protein-like protein	2.90
TC033856	post-rupture	cytoskeleton	CG8213	2.54
TC008606	post-rupture	transmembrane transport	Excitatory amino acid transporter 3-like protein	-2.89
TC011349	post-rupture	cuticle	Chondroitin proteoglycan 2-like protein	-3.32

Supplementary Table 3. ***Tribolium castaneum*** **(*TC*) primer sequences for *in situ* hybridization, RNAi, and RT-qPCR.** Lower case letters in the primer sequences indicate adapter sequences for subsequent amplification with the T7 promoter universal primers, as described[11].

TC gene identifier / primer orientation	sequence	amplicon size cDNA/gDNA [bp]
***in situ* hybridization**		
TC000921 (*Tc-zen1*) / F	ggccgcggTCCCAATTTGAAAACCAAGC	688
TC000921 (*Tc-zen1*) / R	cccggggcCGTTCCACCCTTCCTGATAA	
TC000922 (*Tc-zen2*) / F	ggccgcggAACGCCCCAGTTTTCAACAA	546
TC000922 (*Tc-zen2*) / R	cccggggcCTCATCCTTCACCACCACCT	
**RNAi**		
TC000921 (*Tc-zen1*) / F	ggccgcggTTTGAAAACCAAGCCGTTCT	203 (short fragment)
TC000921 (*Tc-zen1*) / R	cccggggcCGTTGGGGTTGAGTTTCTTG	
TC000921 (*Tc-zen1*) / F	ggccgcggTTTGAAAACCAAGCCGTTCT	682 (long fragment)
TC000921 (*Tc-zen1*) / R	cccggggcCGTTCCACCCTTCCTGATAA	
TC000922 (*Tc-zen2*) / F	ggccgcggCAATGTCGCCGCAATCGACG	250
TC000922 (*Tc-zen2*) / R	cccggggcACACAATTCTTCCCTTGGTA	
**RT-qPCR**		
TC000921 (*Tc-zen1*) / F	TCCACCTTCTGATTGGAACTG	105/161
TC000921 (*Tc-zen1*) / R	CGTTGGGGTTGAGTTTCTTG	
TC000922 (*Tc-zen2*) / F	TCGAAGTGTCCCTCTCAGAAA	101/147
TC000922 (*Tc-zen2*) / R	GGAGGAGGTGTACGCAGTTC	
TC008261 (*Tc-RpS3*) / F	ACCGTCGTATTCGTGAATTGAC	186
TC008261 (*Tc-RpS3*) / R	ACCTCAAAACACCATAGCAAGC	
TC010840-RA / F	TGCGCCTCTCTTCAGTACCT	127/563
TC010840-RA / R	CGCCAGGTAAAGGCATACAC	
TC011665-RA / F	AAGACGCAGCTTTGACCAAT	142/198
TC011665-RA / R	ATCATCATACCGCCCATCAT	
TC005982-RA / F	GTATTGTGTACGCGGGGACT	117/2790
TC005982-RA / R	TTGTTGAAAACCCACCCTCT	
TC006575-RA / F	TTACCATTTGTCCCGAGTCC	144/193
TC006575-RA / R	CGAACTTCGTGTCGCAAATA	
TC014041-RA / F	TATGGCAGCCACAAGAAGC	150/5369
TC014041-RA / R	GTTGGGGTGGTGTCGTAGAT	
TC014143-RA / F	CGAAGACGATAAAGAGGGCTA	123/5213
TC014143-RA / R	TTCATGGCACTATACTGGTTCG	
TC014497-RA / F	GCTCTTCGTTTCACTTGTGG	137/183
TC014497-RA / R	TGCCGTCACTGGTCTCATAC	
TC007162-RA / F	CGTCGCAACCTGTAAGTCTG	148/3480
TC007162-RA / R	TCGTTCATCAGCGTGAAGTC	
TC008606-RA / F	CAAGCTGGCCTCGTCACTAT	134/10182
TC008606-RA / R	ATGCAGTCGCACATCACATT	
TC011349-RA / F	ACCATCGTTACCCTCATTGC	122423
TC011349-RA / R	TCCCTGCATTCGATATAGCC	
TC031481-RA / F	GCACCCCGATAACGGATTA	114/4865
TC031481-RA / R	GGGATTTCACCATTTACTGGA	
TC033856-RA / F	GAAGAGGCCGAAAACTACGA	150/1157
TC033856-RA / R	GCCCCTTTACCACCGACTAT	

Supplementary Table 4. Comparison of alignment statistics by read length. Data are from all the sequenced samples collected during early development. The difference in mapping efficiency of the same sample mapped as 75-bp reads and 100-bp reads is shown. The samples mapped as 75-bp reads have higher mapping efficiency, gaining 9-15% of uniquely mapping reads and only 3-4% of multi-mapping reads. Overall alignment rate represents the sum of uniquely mapping and multi-mapping reads.

	**Unique-mapping [%]**		**Multi-mapping [%]**		**Overall alignment rate [%]**	
**Sample name**	**100 bp**	**75 bp**	**100 bp**	**75 bp**	**100 bp**	**75 bp**
Tc-zen1^{RNAi} 1	55	68	14	17	69	85
Tc-zen1^{RNAi} 2	55	69	13	17	68	86
Tc-zen1^{RNAi} 3	54	64	15	19	69	83
Tc-zen1 WT 1	51	66	14	18	65	84
Tc-zen1 WT 2	54	68	14	17	68	85
Tc-zen1 WT 3	56	67	14	17	70	84
Tc-zen2^{RNAi} 1	57	68	14	17	71	85
Tc-zen2^{RNAi} 1	58	68	14	17	72	85
Tc-zen2^{RNAi} 1	57	68	14	17	71	85
Tc-zen2 WT 1	56	64	15	18	71	82
Tc-zen2 WT 2	58	67	14	17	72	84
Tc-zen2 WT 3	55	66	14	17	69	83
**range**	**51-58**	**64-69**	**13-15**	**17-19**	**65-72**	**82-86**
**difference**	**9-15**		**3-4**		**11-19**	

References

1 McKenna, D. D. *et al.* Genome of the Asian longhorned beetle (*Anoplophora glabripennis*), a globally significant invasive species, reveals key functional and evolutionary innovations at the beetle–plant interface. *Genome Biol.* **17**, 227 (2016).

2 Benoit, J. B. *et al.* Unique features of a global human ectoparasite identified through sequencing of the bed bug genome. *Nat. Commun.* **7**, 10165 (2016).

3 Schoville, S. D. *et al.* A model species for agricultural pest genomics: the genome of the Colorado potato beetle, *Leptinotarsa decemlineata* (Coleoptera: Chrysomelidae). *Scientific reports* **8**, 1931 (2018).

4 Hilbrant, M., Horn, T., Koelzer, S. & Panfilio, K. A. The beetle amnion and serosa functionally interact as apposed epithelia. *eLife* **5**, e13834 (2016).

5 Mackrodt, D. *Etablierung und Funktion maternaler Proteingradienten im Tribolium Blastoderm* Ph.D. (Dr. rer. nat.) thesis, Friedrich-Alexander-Universität Erlangen-Nürnberg, (2016).

6 Panfilio, K. A. Late extraembryonic development and its *zen-RNAi*-induced failure in the milkweed bug *Oncopeltus fasciatus*. *Dev. Biol.* **333**, 297-311 (2009).

7 Dorn, A. Ultrastructure of embryonic envelopes and integument of *Oncopeltus fasciatus* Dallas (Insecta, Heteroptera) I. Chorion, amnion, serosa, integument. *Zoomorphologie* **85**, 111-131 (1976).

8 Lamer, A. & Dorn, A. The serosa of *Manduca sexta* (Insecta, Lepidoptera): ontogeny, secretory activity, structural changes, and functional considerations. *Tissue Cell* **33**, 580-595 (2001).

9 Panfilio, K. A. Extraembryonic development in insects and the acrobatics of blastokinesis. *Dev. Biol.* **313**, 471-491 (2008).

10 Shah, N., Dorer, D. R., Moriyama, E. N. & Christensen, A. C. Evolution of a large, conserved, and syntenic gene family in insects. *G3 (Bethesda)* **2**, 313-319 (2012).

11 Koelzer, S., Kölsch, Y. & Panfilio, K. A. Visualizing late insect embryogenesis: Extraembryonic and mesodermal enhancer trap expression in the beetle *Tribolium castaneum*. *PLoS One* **9**, e103967 (2014).
