# Supplementary material for: Unexpected mutual regulation underlies paralogue functional diversification and promotes epithelial tissue maturation in Tribolium
Source: Commun Biol. 2020 Oct 5;3:552. doi: 10.1038/s42003-020-01250-3 (PMC7536231; doi:10.1038/s42003-020-01250-3)
Supplement: Supplementary file 2 — Description of Additional Supplementary Files [file 42003_2020_1250_MOESM2_ESM.pdf]

Description of Additional Supplementary Files

File Name: Supplementary Data 1
Description: Tables of differentially expressed genes during early development (P_{adj}≤0.01). **(A)** After *Tc-zen1* RNAi compared to wild type, at 6-10 hAEL. **(B)** After *Tc-zen2* RNAi compared to wild type, at 10-14 hAEL. **(C)** Between wild type at 6-10 hAEL and 10-14 hAEL

File Name: Supplementary Data 2
Description: Regulatory targets shared by both *Tc-zen* paralogues with relaxed thresholds (P_{adj}≤0.05). **(A)** Same direction of regulation, n=42. **(B)** Opposite direction of regulation, n=78.

File Name: Supplementary Data 3
Description: Tables of differentially expressed genes during late development (P_{adj}≤0.01). **(A)** After *Tc-zen2* RNAi compared to wild type, at prerupture (48-52 hAEL). **(B)** After *Tc-zen2* RNAi compared to wild type, during rupture (52-56 hAEL). **(C)** Between wild type at 48-52 hAEL and 52-56 hAEL. **(D)** Between *Tc-zen2* RNAi at 48-52 hAEL and 52-56 hAEL. **(E)** Between wild type at 48-52 hAEL and *Tc-zen2* RNAi 52-56 hAEL.

File Name: Supplementary Data 4
Description: Tables of enriched gene ontology (GO) terms for differentially expressed genes after *Tc-zen2* RNAi in late development. **(A)** At prerupture (48-52 hAEL). **(B)** During rupture (52-56 hAEL).

File Name: Supplementary Data 5
Description: Source values and dual plotting (means, individual values) for bar charts in figures.
