# Supplementary material for: Unexpected mutual regulation underlies paralogue functional diversification and promotes epithelial tissue maturation in Tribolium
Source: Commun Biol. 2020 Oct 5;3:552. doi: 10.1038/s42003-020-01250-3 (PMC7536231; doi:10.1038/s42003-020-01250-3)
Supplement: Supplementary file 6 — Reporting Summary [file 42003_2020_1250_MOESM6_ESM.pdf]

nature research

Corresponding author(s): Kristen Panfilio

Last updated by author(s): Aug 11, 2020

Reporting Summary

Nature Research wishes to improve the reproducibility of the work that we publish. This form provides structure for consistency and transparency in reporting. For further information on Nature Research policies, see Authors & Referees and the Editorial Policy Checklist .

Statistics

For all statistical analyses, confirm that the following items are present in the figure legend, table legend, main text, or Methods section.

n/a	Confirmed	
☐	☒	The exact sample size (*n*) for each experimental group/condition, given as a discrete number and unit of measurement
☐	☒	A statement on whether measurements were taken from distinct samples or whether the same sample was measured repeatedly
☐	☒	The statistical test(s) used AND whether they are one- or two-sided<br>*Only common tests should be described solely by name; describe more complex techniques in the Methods section.*
☒	☐	A description of all covariates tested
☐	☒	A description of any assumptions or corrections, such as tests of normality and adjustment for multiple comparisons
☐	☒	A full description of the statistical parameters including central tendency (e.g. means) or other basic estimates (e.g. regression coefficient) AND variation (e.g. standard deviation) or associated estimates of uncertainty (e.g. confidence intervals)
☒	☐	For null hypothesis testing, the test statistic (e.g. *F*, *t*, *r*) with confidence intervals, effect sizes, degrees of freedom and *P* value noted<br>*Give P values as exact values whenever suitable.*
☒	☐	For Bayesian analysis, information on the choice of priors and Markov chain Monte Carlo settings
☒	☐	For hierarchical and complex designs, identification of the appropriate level for tests and full reporting of outcomes
☒	☐	Estimates of effect sizes (e.g. Cohen's *d*, Pearson's *r*), indicating how they were calculated

Our web collection on statistics for biologists contains articles on many of the points above.

Software and code

Policy information about availability of computer code

Data collection	No software was used for data collection
Data analysis	All data analysis software are described in the methods, with version number and citations and/or URL links and date of accession. No custom software was used.

For manuscripts utilizing custom algorithms or software that are central to the research but not yet described in published literature, software must be made available to editors/reviewers. We strongly encourage code deposition in a community repository (e.g. GitHub). See the Nature Research guidelines for submitting code & software for further information.

Data

Policy information about availability of data

All manuscripts must include a data availability statement. This statement should provide the following information, where applicable:

- Accession codes, unique identifiers, or web links for publicly available datasets
- A list of figures that have associated raw data
- A description of any restrictions on data availability

All processed data and analyses generated during this study are included in this published article and its supplementary information files, including source data: Supplementary Data 1-3: tables of differentially expressed genes from all comparisons; Supplementary Data 4: gene ontology (GO) terms for differentially expressed genes after Tc-zen2 RNAi in late development; Supplementary Data 5: source values and dual plotting (means, individual values) for bar charts in figures (Figs. 1D,2G,3A,6B; Supplementary Fig. 8B). The RNA-seq raw read data generated during the current study are available in GenBank (NCBI), under Bioproject accession number PRJNA645519. The paralogue-specific peptide antibodies are available on request from the corresponding author or from the source laboratory.

Field-specific reporting

Please select the one below that is the best fit for your research. If you are not sure, read the appropriate sections before making your selection.

☒ Life sciences ☐ Behavioural & social sciences ☐ Ecological, evolutionary & environmental sciences

For a reference copy of the document with all sections, see nature.com/documents/nr-reporting-summary-flat.pdf

Life sciences study design

All studies must disclose on these points even when the disclosure is negative.

Sample size	Sample sizes of 3 or 4 biological replicates were used for RNA-seq and RT-qPCR, as specified, to allow for statistical comparisons balanced against the labor-intensive nature of generating each biological replicate.
Data exclusions	No data were excluded from analyses; quality control and raw read trimming are described in the methods and follow conventional procedures that are cited.
Replication	As indicated in the manuscript, in addition to the use of direct biological replicates for any one technique, expression data were corroborated across RT-qPCR, RNA-seq, and in situ hybridization methods (mRNA transcript) or across Western blot and immunohistochemistry (protein).
Randomization	Randomization was not applicable in this direct comparison of wild type and RNAi-treated samples.
Blinding	Blinding was not relevant, as all in vitro molecular data were processed with identical pipelines (RNA-seq, RT-qPCR), while embryos used for in situ hybridization were treated in identical fashions (e.g., duration of staining during NBT/BCIP precipitation) within the same experiment.

Reporting for specific materials, systems and methods

We require information from authors about some types of materials, experimental systems and methods used in many studies. Here, indicate whether each material, system or method listed is relevant to your study. If you are not sure if a list item applies to your research, read the appropriate section before selecting a response.

Materials & experimental systems

n/a	Involved in the study	
☐	☒	Antibodies
☒	☐	Eukaryotic cell lines
☒	☐	Palaeontology
☐	☒	Animals and other organisms
☒	☐	Human research participants
☒	☐	Clinical data

Methods

n/a	Involved in the study	
☒	☐	ChIP-seq
☒	☐	Flow cytometry
☒	☐	MRI-based neuroimaging

Antibodies

Antibodies used	Tc-Zen1 and Tc-Zen2 specific peptide antibodies for Tribolium castaneum (Schoppmeier Lab)
Validation	The primary antibodies are described and characterized in the cited Ph.D. thesis from the lab that generated them.

Animals and other organisms

Policy information about studies involving animals; ARRIVE guidelines recommended for reporting animal research

Laboratory animals	Tribolium castaneum, San Bernardino wild type strain
Wild animals	The study did not involve wild animals
Field-collected samples	The study did not involve animals collected from the field
Ethics oversight	No ethical approval or guidance was sought, as no human, animal (vertebrate), or GMO materials were used. The research was conducted in a certified low-risk S1 level laboratory.

Note that full information on the approval of the study protocol must also be provided in the manuscript.
